# Antimicrobial-resistant pathogens in fruits and vegetables from retail and home gardens

**DOI:** 10.1093/sumbio/qvad002

**Published:** 2024-01-17

**Authors:** Afolake Olanbiwoninu, Theresa Awotundun, John Olayiwola, Yinka Somorin

**Affiliations:** Department of Biological Sciences, Ajayi Crowther University, 211212, Oyo town, Oyo State, Nigeria; Department of Biological Sciences, Ajayi Crowther University, 211212, Oyo town, Oyo State, Nigeria; Department of Biological Sciences, Ajayi Crowther University, 211212, Oyo town, Oyo State, Nigeria; School of Natural Sciences, University of Galway, Galway, H91 TK33, Ireland

**Keywords:** fruits, vegetables, antimicrobial resistance, genes, food safety

## Abstract

Fruits and vegetables have been identified as vehicles for the transmission of pathogenic antimicrobial-resistant (AMR) microorganisms. This is of food safety concern, thus requiring continuous surveillance. This study aimed to profile AMR bacteria present in selected fruits and vegetables retailed in markets and from home gardens (HGs) in Oyo, Ogun, and Ekiti states, Nigeria. Watermelon, cucumber, tomato, and garden egg samples were collected and analyzed using standard microbiological procedures. The susceptibility of the isolates to eight antibiotics was determined. Multidrug-resistant isolates were screened for the presence of AMR genes by polymerase chain reaction. Fifty three bacteria were isolated and identified, belonging to the genera *Bacillus, Corynebacterium, Listeria, Aeromonas, Enterobacter, Erwinia, Salmonella, Serratia, Shigella*, and *Vibrio*. Thirty six (67.93%) isolates demonstrated phenotypic resistance to five of the eight antibiotics tested, being the most prevalent pattern observed. *Bla*_TEM_ and *bla*_CTX-M_ were detected in *Salmonella enterica* from retailed tomato, *bla*_TEM_, *bla*_SHV_, *bla_CTX_*_-M_, and *erm*(B) were detected in *Listeria monocytogenes* from retailed watermelon, *bla*_SHV_ and *bla*_CTX-M_ were detected in *Bacillus cereus* from retailed tomato, while *bla*_TEM_, *bla*_SHV_, *bla*_CTX-M_, and *erm*(F) were detected in *Staphylococcus aureus* isolated in garden egg from HG. The presence of multidrug-resistant pathogens in fruits and vegetables could pose a huge food safety and public health risk.

**Sustainability Statement**

There have been considerable efforts on surveillance of antimicrobial resistance (AMR) in bacteria from humans and food-producing animals in recent years in developed countries; however, less attention has been given to other One Health contexts such as the environment and foods, particularly in low- and middle-income countries. This study gives insight into the presence of antimicrobial-resistant pathogens in retailed and home-grown fruits and vegetables in southwestern Nigeria. This will assist in the improvement of hygienic practices at different stages of the value chain of fruits and vegetables, hence addressing Sustainable Development Goal 3: Good Health and Wellbeing.

## Introduction

Antimicrobial resistance (AMR) is a significant threat to public health. The growing challenge of the emergence and spread of AMR has been the subject of several surveys and has been reported by the World Health Organization (WHO) as one of the major health challenges of the 21st century [WHO 2015, Food and Agriculture Organization 2018]. AMR has been recognized as a threat to the world’s sustainability and development efforts, including the United Nations Sustainable Development Goals (SDGs) (Jasovky et al. 2016). It was estimated in 2019 that 1.27 million people died due to antimicrobial-resistant infections, and the global burden associated with antimicrobial-resistant infections across several pathogen–drug combinations was estimated at 4.95 million (Antimicrobial Resistance Collaborators 2022). To mitigate the global challenge of AMR, several strategies are being implemented to reduce exposure to antimicrobial-resistant pathogens and lower the burden of AMR, including antimicrobial stewardship, early, rapid, and accurate diagnosis to inform the treatment of infections, improved public education and awareness, and continuous and holistic surveillance in a One Health context (World Health Organization 2019).

There have been considerable efforts on surveillance of AMR in bacteria from humans and food-producing animals in recent years in developed countries (World Health Organization and the European Centre for Disease Prevention and Control 2022); however, less attention has been given to other One Health contexts such as the environment and foods, particularly in low- and middle-income countries (Ikhimiukor et al. 2022). Agricultural products, including fruits and vegetables, can act as significant reservoirs of antimicrobial-resistant microorganisms (Zahra et al. 2016). Fruits and vegetables are essential for healthy nutrition and are consumed globally due to their numerous health benefits, which include improved mental health (Brookie et al. 2018), reduced cancer risk (Gonzalez et al. 2012, Oyebode et al. 2014), reduced cardiovascular disease mortality (Oyebode et al. 2014), and reduced risk of stroke (Hu et al. 2014), among others. Nonetheless, fruits and vegetables could pose a huge public health risk due to the possibility of being vehicles for transmitting antimicrobial-resistant pathogens to humans (Schwaiger et al. 2011, Ahmed et al. 2019, Jung and Rubin 2020, Oyedele et al. 2020, Saksena et al. 2020). This risk could be heightened based on the recommendation of adopting home gardens (HGs) to promote food security and well-being (Galhena et al. 2013), since fruits and vegetables grown in HGs could come in contact with contaminated inputs (soil, manure, and water), animals (pets, livestock, or wild animals), and humans (Young et al. 2022).

The presence of antimicrobial-resistant bacteria in fruits and vegetables constitutes a huge food safety concern, because they are often consumed raw or minimally processed. Identifying antimicrobial-resistant bacteria in fruits and vegetables would help to understand the risk of exposure of local populations to AMR through foods and would contribute to the development of appropriate strategies to ensure food safety in the value chain of fresh fruits and vegetables from production to consumption. While some studies have reported antimicrobial-resistant pathogens in retailed samples in parts of Nigeria (Adesetan et al. 2013, Owolabi and Ichoku 2013, Adekanle et al. 2015, Olaniyi et al. 2019, Oyedele et al. 2020, Ajiboye and Emmanuel 2021, Ugwu et al. 2022), there is limited information on antimicrobial-resistant pathogens in fruits and vegetables grown in HGs. Therefore, this study aimed to determine the presence of antimicrobial-resistant pathogens in retailed and home-grown fruits and vegetables in southwestern Nigeria.

## Materials and methods

### Sample collection

Fruits and vegetables that grow close to the soil and are minimally processed before consumption were selected for this study. A total of 53 fruit and vegetable samples, including cucumber (*n *= 5), watermelon (*n *= 3), garden egg (*n* = 16), and tomato (*n* = 29), were collected aseptically in sterile bags from different markets and HGs from Oyo, Ogun, and Ekiti states in southwestern Nigeria (Table 1). The number of samples collected from each state is provided in Table S1. The samples were labeled accordingly and transported in sterile plastic bags to the Microbiology Laboratory, Department of Biological Sciences, Ajayi Crowther University, for analyses.

**Table 1.** Distribution of fruit and vegetable samples.

Fruits/vegetables	Retail samples (*n*)	HG samples (*n*)
Cucumber	3	2
Watermelon	2	1
Garden egg	10	6
Tomatoes	15	14

### Microbial isolation and identification

A sharp sterile blade was used to cut out the epicarp and mesocarp of each sample in a sterile tray after disinfecting the surface of each sample. One gram of each sample was weighed into a sterile stomacher bag (Seward, UK) and homogenized using a stomacher (Stomacher 80 Biomaster, Seward, UK) with 9 ml of sterile water for 2 min. The homogenate was serially diluted with sterile water and pour plated on nutrient agar (NA) (HiMedia, India), brain–heart infusion agar (BHIA) (HiMedia, India), MacConkey agar (MCA) (HiMedia, India), Eosin Methylene blue agar (EMBA) (HiMedia, India), and *Salmonella*–*Shigella* agar (SSA) (HiMedia, India) in duplicates. The samples were analyzed for aerobic mesophilic count (NA) and the presence of *Listeria* (BHIA), *Enterobacteriaceae* (MCA), *Escherichia coli* (EMBA), and *Salmonella* and *Shigella* (SSA). All isolated bacteria were identified using conventional techniques such as colonial characteristics, Gram’s staining, and biochemical reactions such as carbohydrate fermentation, urease production, and citrate utilization among others as required following Bergey’s manual of determinative bacteriology (Bergey and Holt 1994).

### Evaluation for amylase and hemolysin production

All isolates were subjected to hemolysis assay and amylase production tests to gain an insight into their pathogenic and fruit spoilage potential, respectively. These tests are, however, preliminary and not definitive determinants of the pathogenicity and spoilage potentials of an organism. The hemolysis assay was achieved by streaking each isolate on freshly prepared blood agar (10% human blood free of antibiotics in 100 ml NA; v/v) and incubated. Afterward, they were grouped into γ, α, and β-hemolytic bacteria. The amylase test was carried out using starch agar (w/v: 0.5% peptone, 0.3% yeast extract, 1.5% agar–agar, 0.2% soluble starch), the isolates were classified into amylase producers and nonamylase producers. Isolates that fell into the category of β-hemolytic and at the same time amylase producers were considered potential pathogens (Oyedele et al. 2020).

### Antimicrobial susceptibility testing

#### Inoculum preparation

Inocula were prepared by standardizing isolates to contain ∼1.5 × 10^8^ CFU/ml based on 0.5 McFarland Standard.

#### Antibiotic susceptibility testing

Antibiotic susceptibility testing was conducted by the disc diffusion method on Mueller–Hinton agar (HiMedia). The antibiotics used against Gram-positive isolates were Ceftazidime (CAZ, 30 μg), Cefuroxime (CRX, 30 μg), Gentamicin (GEN, 10 μg), Ceftriazone (CTR, 30 μg), Erythromycin (ERY, 5 μg), Cloxacillin (CXC, 5 μg), Ofloxacin (OFL, 5 μg), and Amoxicillin/Clavulanate (AUG, 30 μg). The antibiotics used against Gram-negative isolates were Ceftazidime (CAZ, 30 μg), Cefuroxime (CRX, 30 μg), Gentamicin (GEN, 10 μg), Ciprofloxacin (CPR, 5 μg), Ofloxacin (OFL, 5 μg), Amoxicillin/Clavulanate (AUG, 30 μg), Nitrofurantoin (NIT, 300 μg), and Ampicillin (AMP, 10 μg). All antibiotics were from Rapid Labs (UK). The plates were incubated at 37°C for 18–20 h after inoculation with standardized inoculum and placement of the discs. Zones of inhibition were measured in millimeters. Isolates were classified as susceptible, intermediate, or resistant according to the clinical interpretative criteria recommended by the Clinical and Laboratory Standards Institute guidelines (CLSI 2018). The multiple antibiotic resistance (MAR) indices were calculated according to Annapurna and Rashmi (2014). The formula MAR = a/b was applied (where a = the number of antibiotics to which an isolate is resistant and b = the  total number of antibiotics tested).

### Detection of genomic markers of antibiotic resistance

Following the antimicrobial susceptibility testing, bacterial pathogens that were multidrug-resistant were selected to detect the presence of some genomic markers of antibiotic resistance, such as *bla*_TEM_, *bla*_SHV_, *bla*_CTX-M_, *erm*(B), and *erm*(F). These genes were used because a majority of the isolates were resistant to the beta-lactam and erythromycin antibiotics used in this study.

#### DNA extraction

Two ml of overnight suspension cultures of isolates in nutrient broth (at 37°C with shaking) were used for DNA extraction. Briefly, cells were recovered from suspension by centrifugation at 10 000 rpm for 10 min. Thereafter, DNA was extracted from the recovered cell pellets by using the Zymo (ZR) Quick-DNA Miniprep Kit (Zymo Research, USA) following the manufacturer's instructions. The presence of genomic DNA in all prepared isolates was confirmed by 1% agarose gel electrophoresis. The concentrations and purities of the genomic DNA were determined using the NanoDrop spectrophotometer (ThermoFisher, USA).

#### Polymerase chain reaction amplification

The polymerase chain reaction (PCR) mixture consisted of 2.5 μl of 10 × PCR buffer, 1 μl of 25 mM MgCl_2_, 1 μl each of forward and reverse primers, 1 μl of dimethyl sulfoxide (DMSO), 2 μl of 2.5 Mm dNTPs, 0.1 μl of 5 u/μl *Taq* DNA polymerase, and 3 μl of 10 ng/μl DNA. The total reaction volume was made up to 25 μl with nuclease-free water (Gibco, USA). The forward and reverse primers used were previously described by Eguale et al. (2017) and Florez et al. (2014). For the specific gene amplification, the PCR amplification profile consists of initial denaturation at 94ºC for 5 min, followed by 36 cycles of denaturation at 94ºC for 30 s, annealing at 55ºC for 30 s, and elongation at 72ºC for 45 s. These were followed by a final elongation step at 72ºC for 7 min. Amplified fragments were visualized on ethidium bromide-stained 1.5% agarose electrophoresis gels.

## Results

### Characteristics and occurrence of isolates

A total of 146 bacteria were isolated from retail and HG fruits and vegetables; 75 (45.7%) were from retail samples and 71 (43.3%) were from HG samples.

After screening for the amylase and hemolysin production potentials, a total of 53 bacterial isolates were both β-hemolytic and amylase producers, and they were considered potential pathogens. They consisted of 34 (64.2%) from retail samples and 19 (35.8%) from HG samples. *Bacillus cereus* had the highest prevalence in retail (13.2%) and HG (11.3%) samples, occurring in retail cucumber, tomato, and watermelon as well as in garden egg, tomato, and watermelon from HGs. Details of bacterial isolates from the fruits and vegetables are shown in Table 2.

**Table 2.** Bacteria isolated from retail and HG fruits and vegetables.

Fruits/vegetables	Retail samples	HG samples
Cucumber	*Bacillus licheniformis* (1) *Bacillus* spp. (2) *B. cereus* (1) *Bacillus pantotheticus* (1) *Listeria monocytogenes* (1) *C. kutscheri* (2) *Erwinia cacticida* (1) *Aeromonas* sp. (1) *Aeromonas hydrophila* (1)	*Corynebacterium kutscheri* (2) *Bacillus brevis* (1)
Garden egg	*Staphylococcus aureus* (1) *Enterobacter intermedius* (1)	*C. kutscheri* (1) *B. cereus* (2) *S. aureus* (1) *E. cacticida* (1) *Aeromonas* sp. (1)
Tomato	*Corynebacterium xerosis* (1) *B. cereus* (3) *Bacillus* sp. (1) *Bacillus azotoformans* (1) *Shigella flexneri* (1) *Salmonella enterica* (1)	*B. cereus* (3) *L. monocytogenes* (1) *Serratia marcescens* (1) *Serratia fonticola* (1)
Watermelon	*B. cereus* (3) *C. kutscheri* (3) *C. xerosis* (1) *Bacillus* spp. (3) *B. brevis* (1) *L. monocytogenes* (1) *Vibrio* sp. (1)	*C.xerosis* (3) *B. cereus* (1)

### Antibiotic-resistance profile of the bacterial isolates

All isolates of *B. azotoformans, B. brevis, B. cereus, B. licheniformis, B. pantothenticus, Bacillus* spp., *C. kutscheri, C. xerosis, L. monocytogenes*, and *S. aureus* from retail and HG samples were resistant to ceftazidime, cefuroxime, and cloxacillin (Table 3a and b). Among the bacterial isolates from the retail samples, all species isolated were resistant to at least one antibiotic, except *S. flexneri* which was susceptible to all the antibiotics used in this study. A total of 36 (67.93%) isolates demonstrated phenotypic resistance to five out of the eight antibiotics tested being the most prevalent pattern observed. Furthermore, all the bacterial species isolated from HG samples were resistant to at least one antibiotic (Table 3b).

**Table 3.** (a) Antibiotic resistance profile of bacterial isolates from retail fruit and vegetable samples.

		Resistance to antibiotics(n/N)										
S/N	Bacteria	CAZ	CRX	GEN	OFL	AUG	CTR	ERY	CXC	CPR	NIT	AMP
1	*B. azotoformans*	1/1	1/1	0/1	0/1	1/1	0/1	0/1	1/1	−	−	−
2	*B. brevis*	1/1	1/1	0/1	0/1	1/1	0/1	0/1	1/1	−	−	−
3	*B. cereus*	7/7	7/7	0/7	0/7	6/7	7/7	3/7	7/7	−	−	−
4	*B. licheniformis*	1/1	1/1	0/1	0/1	0/1	0/1	0/1	1/1	−	−	−
5	*B. pantotheticus*	1/1	1/1	0/1	0/1	1/1	0/1	0/1	1/1	−	−	−
6	*Bacillus* spp.	6/6	6/6	0/6	0/6	6/6	6/6	0/6	6/6	−	−	−
7	*C. kutscheri*	5/5	5/5	1/5	0/5	5/5	4/5	0/5	5/5	−	−	−
8	*C. xerosis*	2/2	2/2	0/2	0/2	2/2	2/2	2/2	2/2	−	−	−
9	*L. monocytogenes*	2/2	2/2	0/2	0/2	2/2	2/2	1/2	2/2	−	−	−
10	*S. aureus*	1/1	1/1	0/1	0/1	1/1	0/1	1/1	1/1	−	−	−
11	*Aeromonas* sp.	1/1	0/1	0/1	0/1	0/1	−	−	−	0/1	0/1	0/1
12	*A. hydrophila*	1/1	0/1	0/1	0/1	0/1	−	−	−	0/1	0/1	0/1
13	*E. intermedius*	1/1	1/1	0/1	0/1	0/1	−	−	−	0/1	0/1	1/1
14	*E. cacticida*	0/1	1/1	0/1	0/1	0/1	−	−	−	0/1	0/1	0/1
15	*S. flexneri*	0/1	0/1	0/1	0/1	0/1	−	−	−	0/1	0/1	0/1
16	*S. enterica*	1/1	0/1	0/1	1/1	1/1	−	−	−	1/1	0/1	1/1
17	*Vibrio* sp.	1/1	0/1	0/1	0/1	0/1	−	−	−	0/1	0/1	0/1
(b) Antibiotic resistance profile of bacterial isolates from HG fruits and vegetable samples.												
		Resistant isolates (n/N)										
1	*B. brevis*	1/1	1/1	0/1	0/1	1/1	1/1	1/1	1/1	−	−	−
2	*B. cereus*	6/6	6/6	0/6	0/6	6/6	6/6	1/6	6/6	−	−	−
3	*C. kutscheri*	3/3	3/3	0/3	0/3	3/3	3/3	2/3	3/3	−	−	−
4	*C. xerosis*	3/3	3/3	0/3	0/3	2/3	2/3	0/3	3/3	−	−	−
5	*L. monocytogenes*	1/1	1/1	0/1	0/1	1/1	1/1	1/1	1/1	−	−	−
6	*S. aureus*	1/1	1/1	0/1	0/1	1/1	1/1	1/1	1/1	−	−	−
7	*Aeromonas* sp.	1/1	1/1	0/1	0/1	1/1	−	−	−	0/1	0/1	1/1
8	*E. cacticida*	1/1	1/1	0/1	0/1	0/1	−	−	−	0/1	0/1	0/1
9	*S. fonticola*	1/1	1/1	0/1	0/1	0/1	−	−	−	0/1	0/1	0/1
10	*S. marcescens*	0/1	0/1	0/1	0/1	0/1	−	−	−	0/1	1/1	1/1

n/N: number of resistant isolates/number of total isolates tested; -: not tested.

CAZ: Ceftazidime (30 μg); CRX: Cefuroxime (30 μg); GEN: Gentamicin (10 μg); CTR: Ceftriaxone (30 μg); ERY: Erythromycin (5 μg); CXC: Cloxacillin (5 μg); OFL: Ofloxacin (5 μg); CPR: Ciprofloxacin (5 μg); AMP: Ampicillin (10 μg); AUG: Amoxicillin-Clavulanic Acid (30 μg); NIT: Nitrofurantoin (300 μg).

### Antibiotic resistance patterns and MAR indices of bacterial species

Isolates that were nonsusceptible to three or more chemical classes of antibiotics out of the six classes of antibiotics used in this study were classified as multidrug resistant. *B. cereus, C. kutscheri, C. xerosis, L. monocytogenes, S. aureus*, and *S. enterica* from retail samples were multidrug resistant with MAR index ranging from 0.625 to 0.75 (Table 4a). From the HG samples, *B. brevis, B. cereus, C. kutscheri, L. monocytogenes, and S. aureus* were multidrug resistant. They were all resistant to antibiotics from the class cephalosporins, macrolides, and penicillins with a MAR index of 0.75 (Table 4b).

**Table 4.** (a) Antibiotic resistance patterns and MAR index of bacterial species from retail fruits and vegetables.

		AMR pattern		
S/N	Bacteria	Antimicrobial class	Profile of MAR	MAR index
1	*B. cereus*	Cephalosporins, macrolides, and penicillins	CAZ, CRX, CTR, ERY, CXC, and AUG	0.75
2	*C. kutscheri*	Cephalosporins, aminoglycosides, and penicillins	CAZ, CRX, GEN, CTR, CXC, and AUG	0.75
3	*C. xerosis*	Cephalosporins, macrolides, and penicillins	CAZ, CRX, CTR, ERY, CXC, and AUG	0.75
4	*L. monocytogenes*	Cephalosporins, macrolides, and penicillins	CAZ, CRX, CTR, ERY, CXC, and AUG	0.75
5	*S. aureus*	Cephalosporins, macrolides, and penicillins	CAZ, CRX, ERY, CXC, and AUG	0.625
6	*S. enterica*	Cephalosporins, fluoroquinolones, and penicillins	CAZ, CPR, OFL, AUG, and AMP	0.625
(b) Antibiotic-resistance patterns and MAR index of bacterial species from HG fruits and vegetables.				
1	*B. brevis*	Cephalosporins, macrolides, and penicillins	CAZ, CRX, CTR, ERY, CXC, and AUG	0.75
2	*B. cereus*	Cephalosporins, macrolides, and penicillins	CAZ, CRX, CTR, ERY, CXC, and AUG	0.75
3	*C. kutscheri*	Cephalosporins, macrolides, and penicillins	CAZ, CRX, CTR, ERY, CXC, and AUG	0.75
4	*L. monocytogenes*	Cephalosporins, macrolides, and penicillins	CAZ, CRX, CTR, ERY, CXC, and AUG	0.75
5	*S. aureus*	Cephalosporins, macrolides, and penicillins	CAZ, CRX, CTR, ERY, CXC, and AUG	0.75

CAZ: Ceftazidime (30 μg); CRX: Cefuroxime (30 μg); GEN: Gentamicin (10 μg); CTR: Ceftriaxone (30 μg); ERY: Erythromycin (5 μg); CXC: Cloxacillin (5 μg); OFL: Ofloxacin (5 μg); CPR: Ciprofloxacin (5 μg); AMP: Ampicillin (10 μg); AUG: Amoxicillin-Clavulanic Acid (30 μg); NIT: Nitrofurantoin (300 μg).

### Detection of antibiotic resistance genes in selected isolates

Pathogenic bacteria that were multidrug resistant and had a high prevalence record in foodborne illnesses, were selected for the detection of genomic markers of antibiotic resistance such as *bla*_TEM_, *bla*_SHV_, *bla*_CTX-M_, *erm*(B), and *erm*(F). The four selected bacterial pathogens were *S. enterica, L. monocytogenes, B. cereus* from retail samples, and *S. aureus* from the HG sample. *Bla*_TEM_ and *bla*_CTX-M_ were detected in *S. enterica* isolated from retailed tomato, which were resistant to five of the eight antibiotics tested*. Bla*_SHV_ and *bla*_CTX-M_ were present in *B. cereus* isolated from retailed tomatoes, which were resistant to six antibiotics. All beta-lactamase genes (*bla*_TEM_, *bla*_SHV_, and *bla*_CTX-M_) and the erythromycin resistance gene, *erm*(B) tested were detected in *L. monocytogenes* isolated from retailed watermelon, which were resistant to six antibiotics. All beta-lactam resistance genes, bla_TEM_, bla_SHV_, and bla_CTX-M_, and the erythromycin resistance gene, *erm*(F), were present in *S. aureus* isolated from garden eggs from the HG, which was resistant to six antibiotics.

## Discussion

This study demonstrates that the retail fruits and vegetables had more bacterial isolate contamination (34) than the HG samples (19). This might have occurred because there were more retail samples (*n* = 30) compared to HG samples (*n* = 23), and this could have contributed to the higher number of bacterial isolates from the retail samples. Bacteria from HG fruits and vegetables were more resistant to the antibiotics tested than retail samples. The bacterial isolates showed varying patterns of resistance to the antibiotics used in this study. The bacteria from retail samples were highly resistant to cloxacillin (100%), ceftazidime (94.1%), cefuroxime (85.3%), ceftriaxone (77.8%), and amoxicillin/clavulanate (76.5%) but showed less resistance to ampicillin (28.6%), erythromycin (25.9%), ciprofloxacin (14.3%), gentamicin (2.9%), and ofloxacin (2.9%). Isolates from retail samples tested against nitrofurantoin were all sensitive to the antibiotics. Bacteria from HG samples also followed the same pattern as retail samples, as they were highly resistant to cloxacillin (100%), ceftazidime (94.7%), cefuroxime (94.7%), ceftriaxone (93.3%), amoxicillin/clavulanate (79.0%), and ampicillin (50.0%) but showed less resistance to erythromycin (40.0%) and nitrofurantoin (25.0%). All isolates from HG samples were sensitive to gentamicin, ofloxacin band ciprofloxacin. Six of the 17 bacterial species from retail samples were *B. cereus, C. kutscheri, C. xerosis, L. monocytogenes, S. aureus*, and *S. enterica* were multidrug resistant. From the HG samples, five of the 10 bacterial species isolated, i.e. *B. brevis, B. cereus, C. kutscheri, L. monocytogenes*, and *S. aureus* were multidrug resistant. The fact that higher numbers of antibiotic-resistant bacteria were associated with the HG and retail samples in this present study is worrisome and suggests that fruits and vegetables may contribute to the transmission of antibiotic-resistant bacteria.

This study is similar to previous reports that have shown the presence of antibiotic-resistant bacteria in fruits and vegetables. Ieren et al. (2013) reported the occurrence of *Listeria* spp., which are multidrug resistant in vegetables sold in Zaria, Nigeria. Oyedele et al. (2020) isolated *Shigella* spp. and *Enterobacter* spp. among other bacterial species from retail fresh-cut ready-to-eat fruits in southwestern Nigeria. The occurrence of *Bacillus* spp., *Corynebacterium* spp., *Listeria monocytogenes, S. aureus*, and *Salmonella* spp. in fruits and vegetables as reported in this study conforms with the reports of Ajiboye and Emmanuel (2021), who reported the presence of *Enterobacter, Salmonella, Shigella, Staphyloccocus*, etc., which were resistant to several antibiotics. Fabiyi et al. (2020) also reported the isolation of different species of *Salmonella* from fruits, which were 100% susceptible to ofloxacin and ciprofloxacin; this result conforms to the findings of this study. Furthermore, Adesetan et al. (2013) reported the occurrence of *B. cereus* in street-vended watermelon in Ogun state, Nigeria; however, unlike the current study, their isolates were susceptible to ceftriaxone and cloxacillin. *Bacillus cereus* isolated from both retail and HG watermelon analyzed in this study were resistant to ceftriaxone and cloxacillin.

The contamination of retail fruits and vegetables might have occurred at any stage along the value chain, from the farm (through soil, seeds, manure, agrochemicals, farm equipment, irrigation equipment, and irrigation water) to the retailer (through handler’s hygiene and storage environmental conditions) (Somorin et al. 2023). The occurrence of bacterial pathogens such as *B. cereus, S. enterica, Corynebacterium* sp., and *L. monocytogenes* in ready-to-eat fruits and vegetables from both retail and HG sources may be a result of transmission from irrigation water, manure, or soil. Obi (2014) isolated *Bacillus* spp., *Salmonella*, and *Shigella* from soil treated with poultry manure and vegetables grown on the soil. In a study carried out on vegetables and the river water used for irrigation of the vegetables by Obinna and Nduchukwuka (2016), *Salmonella* spp. were isolated from both the irrigation water and the vegetables revealing the possibility of contamination of the vegetables from the irrigation water.

This study observed that the beta-lactamase gene *bla*_CTX-M_ was detected in all selected isolates (*S. enterica, L. monocytogenes, B. cereus*, and *S. aureus*) and was more prevalent compared to *bla*_TEM_ and *bla*_SHV_. Furthermore, *erm*(B) was detected in *L. monocytogenes* from retail watermelon and *erm*(F) in *S. aureus* from the HG garden egg. This is similar to Ritcher et al. (2020), who reported *bla*_CTX-M_ followed by *bla*_TEM_ and *bla*_SHV_ dominated *Enterobacteriaceae* isolated from selected commercial spinach. Worldwide, *bla*_TEM_, *bla_S_*_HV_, and *bla*_CTX-M_ are the major extended-spectrum beta-lactamases detected in clinical and agricultural settings, including fruits and vegetables (Njage and Buys 2014, Zhang et al. 2015, Ye et al. 2017). The detection of *erm*(B) in *L. monocytogenes* and *erm*(F) in *S. aureus* has also been reported by Miyebi et al. (2018) and Florez et al. (2014) from lettuce and cheese, respectively. The detection of these genes in fruits and vegetables raises questions concerning food safety. The limitation of our study was that the detection of antibiotic-resistant gene markers was not performed on all AMR isolates; only a few of the isolates were tested due to resource and budget constraints. Other isolates that were not tested in this study have been reported to harbor AMR genes. For example, Trocado et al. (2021) found *b la*_shv_, *bla*_TEM_, and *bla*_CTX-M_ among other genes in the *Enterobacteriaceae* family isolated from fresh fruits.

Hygienic practices at all stages, from farm to retail, are essential to prevent contamination of fruits and vegetables with antibiotic-resistant bacteria (Salamandane et al. 2020). Although the impact of different stages of the value chain of commercially cultivated and home-grown fruits and vegetables on microbiological contamination was not investigated in this present study, it is important to understand the critical control points to define appropriate interventions. Hence, there is a need for further studies investigating the complex value chain for fresh fruits and vegetables. Furthermore, regular antimicrobial susceptibility surveillance in other types of fruits and vegetables is needed to understand the full picture of dietary exposure to AMR bacteria through fruits and vegetables and to track any changes in resistance patterns.

## Conclusion

This study revealed that the analyzed fruits and vegetables were contaminated with diverse pathogenic and antibiotic-resistant microorganisms harboring antibiotic-resistance genes. Contamination was observed in both retail and HG samples; however, retail samples were more contaminated than samples from HGs. Due to their consumption either raw or minimally processed, contamination of fruits and vegetables with AMR pathogens poses a food safety risk and raises a major public health concern, hence requiring continuous surveillance and appropriate interventions to mitigate them.

## Supplementary Material

qvad002_Supplemental_File

## Data Availability

The data underlying this article are available in the article.

## References

[bib1] Adekanle MA, Effedua HI, Oritogun KS et al. A study of microbial analysis of fresh fruits and vegetables, in Sagamu markets South-West, Nigeria. Agrosearch. 2015;15:1–12. 10.4314/agrosh.v15i2.1

[bib2] Adesetan TO, Egberongbe HO, Ilusanya OAFM et al. Antimicrobial sensitivity of bacterial isolates from street vended fruits in Ijebu area of Ogun state, Nigeria. Int Res J Microbiol. 2013;4:220–5.

[bib3] Ahmed S, Siddique MA, Rahman M et al. A study on the prevalence of heavy metals, pesticides, and microbial contaminants and antibiotics resistance pathogens in raw salad vegetables sold in Dhaka, Bangladesh. Heliyon. 2019;5:e01205 10.1016/j.heliyon.2019.e01205. 30805565 PMC6374544

[bib4] Ajiboye AE, Emmanuel TO. Assessment of bacterial contamination in ready-to-eat fruits and vegetables sold at Oja-Oba Market, Ilorin, Nigeria. Afr J Biomed Res. 2021;24:203–9.

[bib5] Annapurna YVS, Rashmi S. Multi drug resistance and MAR index among bacteria associated with fruits and vegetables. Int J Biopharm Res. 2014;3:182–5.

[bib6] Antimicrobial Resistance Collaborators. Global burden of bacterial antimicrobial resistance in 2019: a systematic analysis. Lancet North Am Ed. 2022;399:629–55. 10.1016/S0140-6736(21)02724-0 PMC8841637 35065702

[bib7] Bergey DH, Holt JG. Bergey's Manual of Determinative Bacteriology. 9th edn. Baltimore, MD: Williams & Wilkins, 1994.

[bib8] Brookie KL, Best GI, Conner TS. Intake of raw fruits and vegetables is associated with better mental health than intake of processed fruits and vegetables. Front Psychol. 2018;9:47. 10.3389/fpsyg.2018.00487. 29692750 PMC5902672

[bib9] CLSI (Clinical and Laboratory Standards Institute). Performance Standards for Antimicrobial Susceptibility Testing. Wayne, PA: Clinical and Laboratory Standards Institute. CLSI M100-S28, 2018.

[bib10] Eguale T, Birungi J, Asrat D et al. Genetic markers associated with resistance to beta-lactam and quinolone antimicrobials in non-typhoidal *Salmonella* isolates from humans and animals in central Ethiopia. Antimicrob Resist Infect Control. 2017;6:13. 10.1186/s13756-017-0171-6. 28105330 PMC5240271

[bib11] Fabiyi WA, Fagbola OO, Oloke JK et al. Prevalence of *Salmonella* species contamination rate in some selected edible fruits in Osogbo, Osun state, Southern west, Nigeria. Arch Sci Technol. 2020;1:181–97. https://doi.org/10.xxx

[bib12] Florez AB, Alegria A, Rossi F et al. Molecular identification and quantification of tetracycline and erythromycin resistance genes in Spanish and Italian retail cheeses. BioMed Res Int. 2014;2014:1–10. 10.1155/2014/746859. PMC4180643 25302306

[bib13] Food and Agriculture Organization. Antimicrobial Resistance and Foods of Plant Origin. Summary report of an FAO meeting of experts. Rome: FAO Antimicrobial Resistance Working Group. 2018.

[bib14] Galhena DH, Freed R, Maredia KM. Home gardens: a promising approach to enhance household food security and wellbeing. Agric Food Secur. 2013;2:8. 10.1186/2048-7010-2-8

[bib15] Gonzalez CA, Lujan-Barroso L, Bueno-de-Mesquita HB et al. Fruit and vegetable intake and the risk of gastric adenocarcinoma: a reanalysis of the European Prospective Investigation into Cancer and Nutrition (EPIC-EURGAST) study after a longer follow-up. Int J Cancer. 2012;131:2910–9. 10.1002/ijc.27565. 22473701

[bib16] Hu D, Huang J, Wang Y et al. Fruits and vegetables consumption and risk of stroke: a meta-analysis of prospective cohort studies. Stroke. 2014;45:1613–9. 10.1161/STROKEAHA.114.004836. 24811336

[bib17] Ieren II, Bello M, Kwaga JKP. Occurrence and antibiotic resistance profile of *Listeria monocytogenes* in salad vegetables and vegetable salads sold in Zaria, Nigeria. African J Food Sci. 2013;7:334–8. https://doi.org/10.5897/AJFS2013. 1036

[bib18] Ikhimiukor OO, Odih EE, Donado-Godoy P et al. A bottom-up view of antimicrobial resistance transmission in developing countries. Nat Microbiol. 2022;7:757–65. 10.1038/s41564-022-01124-w 35637328

[bib19] Jasovky D, Littmann J, Zorzet A et al. Antimicrobial resistance: a threat to world's sustainable development. Ups J Med Sci. 2016;121:159–64. 10.1080/03009734.2016.1195900. 27416324 PMC4967260

[bib20] Jung D, Rubin JE. Identification of antimicrobial resistant bacteria from plant-based food products imported into Canada. Int J Food Microbiol. 2020;319:108509. 10.1016/j.ijfoodmicro.2020.108509. 31945714

[bib21] Miyebi JI, Daniel EO, Ezenya CC. *Listeria* species as contaminant of lettuce and its resistant genes in Benin City, Nigeria. J Biotechnol. 2018;35:151–8. 10.4314/njb.v35i2.18.

[bib22] Njage PMK, Buys EM. Pathogenic and commensal *Escherichia coli* from irrigation water show potential in transmission of extended spectrum and *AmpC* beta-lactamases determinants to isolates from lettuce. Microb Biotechnol. 2014;8:462–73. 10.1111/1751-7915.12234. 25488608 PMC4408178

[bib23] Obi CN. Bacteriological assessment of vegetables cultivated in soils treated with poultry manure and the manure treated soil samples. Am J Microbiol Res. 2014;2:189–200. 10.12691/ajmr-2-6-5

[bib24] Obinna CN, Nduchukwuka D. Antibiogram of bacteria species isolated from vegetables in Ado-Odo Ota, Nigeria. J Biol Sci. 2016;16:188–96. 10.3923/jbs.2016.188.196.

[bib25] Olaniyi FT, Akaniro IR, Oguh CE et al. Characterization and antimicrobial resistance profile of pathogenic bacteria isolated from freshly sold *Amaranthus viridis* in Ile-Ife, Southwest Nigeria. J Adv Microbiol. 2019;18:1–8. 10.9734/jamb/2019/v18i130152.

[bib26] Owolabi JB, Ichoku CK. Evaluation of antibiotic resistance pattern of Gram-positive Bacilli isolated from ready-to-eat vegetables in Ota Metropolis, Nigeria. Covenant J Phys Life Sci. 2013;2(2):138–149.

[bib27] Oyebode O, Gordon-Dseagu V, Walker A et al. Fruit and vegetable consumption and all-cause, cancer and CVD mortality: analysis of health survey for England data. J Epidemol Community Health. 2014;68:856–62. 10.1136/jech-2013-203500. PMC4145465 24687909

[bib28] Oyedele OA, Kuzamani KY, Adetunji MC et al. Bacteriological assessment of tropical retail fresh-cut ready-to-eat fruits in south-western Nigeria. Sci Afr. 2020;9:e00505. 10.1016/j.sciaf.2020.e00505

[bib29] Ritcher L, du Plessis EM, Duvenage S et al. Occurrence, phenotypic and molecular characterization of extended-spectrum- and *AmpC*-beta-lactamase producing Enterobacteriaceae isolated from selected commercial spinach supply chains in South Africa. Front Microbiol. 2020;11:638. 32351477 10.3389/fmicb.2020.00638 PMC7176360

[bib30] Saksena R, Malik M, Gaind R. Bacterial contamination and prevalence of antimicrobial resistance phenotypes in raw fruits and vegetables sold in Delhi, India. J Food Saf. 2020;40:e12739. 10.1111/jfs.12739.

[bib31] Salamandane C, Fonseca F, Afonso S et al. Handling of fresh vegetables: knowledge, hygienic behavior of vendors, public health in maputo markets, Mozambique. Int J Environ Res Public Health. 2020;17:6302. 10.3390/ijerph17176302 32872524 PMC7504209

[bib32] Schwaiger K, Helmke K, Holzel CS et al. Antibiotic resistance in bacteria isolated from vegetables with regards to marketing stage (farm vs. supermarket). Int J Food Microbiol. 2011;148:191–6. 21700353 10.1016/j.ijfoodmicro.2011.06.001

[bib33] Somorin YM, Akanni GB, Anyogu A. Microbiological safety and antimicrobial resistance in fresh produce production in Africa. In: Antimicrobial Research and One Health in Africa. Abia ALK, Essack SY (eds.), Springer, Cham 2023, ISBN: 978-3-031-23795-9.Tj

[bib34] Trocado ND, de Moraraes MS, Aveleda L et al. Phenotypic and genotypic detection of antibiotic-resistant bacteria in fresh juices from a public hospital in Rio de Janeriro. Arch Microbiol. 2021;203:1471–5. 10.1007/s00203-020-02139-9. 33398401

[bib35] Ugwu CN, Ezeibe EN, Eze CC et al. Antibacterial susceptibility and biofilm-forming ability of foodborne pathogens isolated from minimally processed fruits and vegetables obtained from Southeastern Nigeria. Trop J Nat Prod Res (TJNPR). 2022;6:422–32. 10.26538/tjnpr/v6i3.20

[bib36] WHO Regional Office for Europe and European Centre for Disease Prevention and Control. Antimicrobial Resistance Surveillance in Europe 2022–2020 Data. Copenhagen: WHO Regional Office for Europe, 2022.

[bib37] WHO. Global Strategy on People-Centered and Integrated Health Services. Geneva: World Health Organization. 2015.

[bib38] World Health Organization (WHO). Turning plans into action for antimicrobial resistance (AMR). Working paper 2.0: implementation and coordination. Geneva. (WHO/WSI/AMR/2019.2). Licence: CC BY-NC-SA 3.0 IGO, 2019.

[bib39] Ye Q, Wu Q, Zhang S et al. Antibiotic-resistant extended beta-lactamase- and plasmid-mediated *AmpC*-producing *Enterobacteriaceae* isolated from retail food products and the pearl river in Guangzhou. China. Front Microbiol. 2017;8:96. 10.3389/fmicb.2017.00096. 28217112 PMC5289952

[bib40] Young CCW, Karmacharya D, Bista M et al. Antibiotic resistance genes of public health importance in livestock and humans in an informal urban community in Nepal. Sci Rep. 2022;12:13808. 10.1038/s41598-022-14781-y 35970981 PMC9378709

[bib41] Zahra SA, Guizani N, Al-Sadi MA et al. Ingesting antibiotic resistant bacteria along with fresh fruits and vegetables and what are the possible consequences. J Food Process Technol. 2016;7(6):54.

[bib42] Zhang H, Zhou Y, Guo S et al. Multidrug resistance found in extended-spectrum beta-lactamase-producing *Enterobacteriaceae* from rural water reservoirs in Guantao, China. Front Microbiol. 2015;6:267. 25873918 10.3389/fmicb.2015.00267 PMC4379920

