# Supplementary material for: Antimicrobial-resistant pathogens in fruits and vegetables from retail and home gardens
Source: Sustain Microbiol. 2024 Jan 17;1(1):qvad002. doi: 10.1093/sumbio/qvad002 (PMC13375307; doi:10.1093/sumbio/qvad002)
Supplement: Supplementary file 1 [file qvad002_Supplemental_File.docx]

Table S1. Collection of samples per State

| Fruits/vegetables | Number of samples per state | | |
| --- | --- | --- | --- |
|  | Ekiti | Ogun | Oyo |
| Cucumber | 2 | 1 | 2 |
| Garden egg | 4 | 6 | 6 |
| Tomato | 7 | 10 | 12 |
| Watermelon | 1 | 1 | 1 |
